# Process evaluation of PrEP implementation in Kenya: adaptation of practices and contextual modifications in public HIV care clinics

**DOI:** 10.1002/jia2.25799

**Published:** 2021-09-08

**Authors:** Elizabeth M. Irungu, Josephine Odoyo, Elizabeth Wamoni, Elizabeth A. Bukusi, Nelly R. Mugo, Kenneth Ngure, Jennifer F. Morton, Kenneth K. Mugwanya, Jared M. Baeten, Gabrielle O'Malley, for the Partners Scale‐Up Project Team

**Affiliations:** ^1^ Department of Global Health University of Washington Seattle Washington USA; ^2^ Department of Epidemiology University of Washington Seattle Washington USA; ^3^ Department of Medicine University of Washington Seattle Washington USA; ^4^ Department of Obstetrics and Gynecology University of Washington Seattle Washington USA; ^5^ Centre for Clinical Research Kenya Medical Research Institute Nairobi Kenya; ^6^ Centre for Microbiology Research Kenya Medical Research Institute Nairobi Kenya; ^7^ School of Public Health Jomo Kenyatta University of Agriculture and Technology Nairobi Kenya; ^8^ Gilead Sciences Foster City CA USA

**Keywords:** adaptation, PrEP continuation, PrEP implementation, PrEP initiation, PrEP integration, public HIV care clinics

## Abstract

**Introduction:**

In Africa, oral pre‐exposure prophylaxis (PrEP) is largely provided via over‐burdened public HIV care clinics. Successfully incorporating PrEP services into these clinics may require adaptations to practices outlined in national implementation guidelines and modifications to routine existing service delivery. We aimed to describe adaptations made by public HIV clinics in Kenya to integrate PrEP delivery into existing services.

**Methods:**

The Partners Scale‐Up Project aimed to catalyse integration of PrEP in 25 public HIV care clinics. Between May and December 2018, we conducted qualitative interviews with health providers and documented clinic observations in technical assistance (TA) reports to understand the process of PrEP service integration. We analysed 36 health provider interview transcripts and 25 TA reports to identify clinic‐level adaptations to activities outlined in Kenyan Ministry of Health PrEP guidelines and modifications made to existing service delivery practices to successfully incorporate PrEP services. Identified adaptations were reported using the expanded framework for reporting adaptations and modifications (FRAME).

**Results:**

All clinics (*n* = 25) performed HIV testing, HIV risk assessment, PrEP education and adherence counselling as stipulated in the guidelines. Most clinics initiated clients on PrEP without creatinine testing if otherwise healthy. While monthly refill appointments are recommended, a majority of clinics issued PrEP users two to three months of pills at a time. Clinics also implemented practices that had not been specified in the guidelines including incorporating PrEP‐related topics into routine health talks, calling clients with missed PrEP appointments, discussing PrEP service delivery in regular staff meetings, ‘fast‐tracking’ PrEP clients and dispensing PrEP in clinic rooms rather than at clinic‐based pharmacies. PrEP initiation numbers were highest among clinics that did not require creatinine testing, conducted peer on‐the‐job PrEP training and those that discussed PrEP delivery in their routine meetings. Above‐average continuation was observed among clinics that discussed PrEP in their routine meetings, dispensed PrEP in clinic rooms and offered PrEP at nonregular hours.

**Conclusions:**

Health providers in public HIV care clinics instituted practices and made innovative adaptations to PrEP delivery to reduce barriers for clients and staff. Encouraging clinic level adaptations to national implementation guidelines will facilitate scale‐up of PrEP delivery.

## 1 INTRODUCTION

The World Health Organization issued a strong recommendation for use of oral pre‐exposure prophylaxis (PrEP), a highly potent HIV prevention intervention that has the potential to reduce HIV incidence markedly if used effectively [1]. Many countries in Africa, the region disproportionately affected by HIV, have adopted this recommendation and begun implementing PrEP programmes [2, 3]. However, despite close to half of all global PrEP initiations being in Africa, scale‐up in the region remains sub‐optimal [4, 5].

Integrating PrEP services into existing programmes may increase access for people at risk of HIV, while promoting the best use of available resources [6, 7, 8]. In theory, public HIV care clinics in Africa are an opportune venue for introduction and integration of PrEP services; clinic staff can identify uninfected partners of their clients living with HIV, have expertise in HIV testing and prescribing antiretroviral drugs and have established ART supply logistics [9]. However, these clinics are typically overburdened, short‐staffed and have associated stigma, which may hinder PrEP implementation efforts. Mitigating these constraints may necessitate adapting activities detailed in guidelines and instituting modifications to routine practices in the clinics [10, 11, 12].

Moving evidence‐based interventions (EBIs) from research settings to scaled implementation requires adaptations of the EBI to better fit within the contextual realities of those delivering the intervention, the needs of the local target population, or to respond to unanticipated challenges [13, 14]. In addition, when EBIs are integrated into existing programmes that were developed to deliver other interventions, modifications to clinic service delivery practices are necessary to make the new programme fit. Unfortunately, the description of what and why modifications are made is infrequently documented and shared in peer reviewed literature, thus limiting the dissemination of learnings from real‐world settings [14]. The expanded framework for reporting adaptations and modifications (FRAME) provides an outline for characterizing modifications to interventions and has been used to describe adaptations to interventions in various fields in health [15, 16, 17, 18, 19, 20]. This paper uses the FRAME to describe adaptations made by public HIV care clinics in Kenya to integrate the delivery of PrEP with the intention of sharing lessons learnt for others interested in scaling PrEP services.

### 1.1 Context

In 2018, approximately 1.4 million adults were living with HIV and in the same year, an estimated 36,000 adults acquired HIV in Kenya [21]. The majority of people living with HIV in Kenya receive HIV care services free of charge in public HIV care clinics [22, 23, 24]. The clinics are staffed by clinicians, nurses, pharmacy staff, laboratory technicians, counsellors and data clerks. In 2016, Kenya revised the national HIV care and treatment guidelines and recommended PrEP as an additional HIV prevention intervention for people at risk of acquiring HIV [25]. By July 2020, Kenya had one of the largest PrEP programmes in Africa with more than 60,000 individuals having initiated PrEP [5, 26].

### 1.2 Guidelines for PrEP delivery

According to national guidelines, individuals eligible to use PrEP are those who are HIV uninfected and who report having multiple sex partners, having sex partners living with HIV and not virally suppressed, having sex partners of unknown HIV status, have had a recent sexually transmitted infection (STI), have no or inconsistent condom use, engage in transactional sex, have recurrent use of postexposure prophylaxis or engage in recreational drug use [25]. At PrEP initiation visits, behavioural risk assessment, risk reduction counselling and HIV testing should be performed. Clients should be evaluated to identify symptoms of acute HIV infection, sexually transmitted infections and presence of known contraindications to PrEP medication before a prescription is issued. Creatinine testing is recommended at baseline, but absence of test results should not delay PrEP initiation. Clients should have a clinical review after one month of PrEP use and every three months thereafter with monthly refill visits in between. Quarterly follow‐up visits should include a rapid HIV test, an assessment of acute HIV symptoms, an evaluation of ongoing need for PrEP, medication side effects and medication adherence. Initiation and follow‐up visits should be documented in a clinical encounter form (CEF).

## 2 METHODS

The Partners Scale‐Up Project was a prospective, implementation study conducted in 25 high‐volume public HIV care clinics in western and central Kenya between January 2017 and December 2020. Selected clinics had an average of 3600 people living with HIV engaged in care. The project aimed to catalyse integration of PrEP in these clinics using existing facility infrastructure and personnel capacity [27]. Health providers were trained on PrEP delivery by project staff, and clinics began delivering PrEP services shortly thereafter [28]. The PrEP training was conducted over two days using a curriculum developed by the Kenya Ministry of Health, in collaboration with stakeholders, including the study team and facilitated by project staff [29]. Pre‐ and post‐training assessments were done to assess PrEP knowledge. Project staff provided routine ongoing technical assistance (TA) and mentorship to providers. Each technical advisor provided support for up to four clinics and visited clinics at least twice a month in the first year and monthly or less frequently thereafter as facilities became comfortable with PrEP delivery. Best practices observed by technical advisors were shared across clinics. At the end of each TA visit, reports that detailed clinic observations and conversations with clinic staff were completed. The report template prompted a description of successes and challenges with demand creation, identification of PrEP clients, PrEP service delivery, workforce, commodities and client retention (see Supporting information file S1). Approximately one year into the project, a summary TA report was generated for each clinic. This analysis is based on two data sources: summary TA reports and key informant interviews (KIIs).

Between May and December 2018, we interviewed healthcare providers using semi‐structured interview guides to elicit details on how PrEP services were integrated into routine clinic practice (see Supporting information file S2). We purposively sampled different cadres, such as clinical officers, nurses, social workers and counsellors from the 25 clinics, to capture different perspectives. Interviews were conducted in either English or Kiswahili depending on the interviewee's preference. Interviews were audio‐recorded, translated to English where necessary and transcribed by the interviewer. Average interview duration was 45 minutes.

Interview transcripts and TA summary reports were analysed in Dedoose (Sociocultural Research Consultants LLC, Los Angeles, CA). An initial codebook was developed by two senior members of the research team, with codes based on the national PrEP delivery guidelines and investigator knowledge of clinical practices at the public HIV clinics. Additional codes related to reasons and outcomes of adaptations were added inductively as initial transcripts, and TA reports were reviewed and coded. The first eight interview transcripts and six TA reports were dual coded by at least two members of the study team, and differences in coding were discussed until consensus was reached. The final codebook was applied to all transcripts and TA reports by one member of the study team and reviewed by another. Questions or disagreements on code application were resolved via consensus.

In order to assess and quantify adherence to PrEP implementation guidelines, we created three categories: (i) *implemented all the time* if all interviews and TA reports for a particular clinic indicate that the activity was implemented; (ii) *implemented some of the time* if some interviews and TA reports for a particular clinic indicate that the activity was not always implemented; (iii) *not implemented* if all interviews and TA reports for a particular clinic indicate that the activity was not implemented. In addition, we categorized clinics as having made modifications to their existing programme practices if any of the interviews and TA reports of a particular clinic indicated that there had been a change at any time. The coded excerpts of text were reviewed, and the categories described above were applied.

We conducted descriptive analysis to determine the frequency with which clinics adhered to activities as stipulated in the guidelines and modified their routine practice. We also compared mean monthly PrEP initiations and continuation rates as of December 2018 between clinics making adaptations to PrEP guidelines and those instituting changes to routine HIV programme practices compared to those that did not. Programmatic data for PrEP initiation and continuation were obtained from abstracted CEF records of individuals receiving PrEP services in participating clinics. The records were abstracted by trained project staff. PrEP initiation was defined as documentation on the CEF of having received a PrEP prescription. Month 6 PrEP continuation was defined as the proportion of people eligible for a visit within six months of PrEP initiation who had a documented PrEP refill within the month 6 visit window (i.e., the period between 15 days before the expected month 6 visit date to 15 days before the next expected visit date).

We used the FRAME to report adaptations [20]. Elements of this framework include a description of what was adapted, the nature and timing of the adaptation, whether the adaptation was planned or not, who determined that the adaptation should be made, for whom it was made, whether it was fidelity‐consistent and the goals of the adaptation. We describe observed adaptations and report on elements that are relevant to our project. All adaptations were made after PrEP implementation had begun in clinics. We determined adaptations to be fidelity‐consistent if they did not remove core elements of PrEP delivery including HIV testing before PrEP initiation, and quarterly thereafter, eligibility assessment and issuance of PrEP prescriptions and dispensing the medication. We present results in a tabular form. Finally, we used thematic content analysis to identify overarching motivations for adaptations and modifications.

### 2.1 Ethics statement

The Scientific and Ethics Review Unit of the Kenya Medical Research Institute and the Human Subjects Division of the University of Washington approved this programme implementation evaluation protocol enabling analysis of de‐identified programmatic data. Written informed consent was provided by all interviewed participants.

## 3 RESULTS

We interviewed 36 providers of whom 56% were female. Their median age was 35 years (interquartile range (IQR) 23 to 65) and they had worked in healthcare for a median duration of 56 months (IQR 4 to 204). Of those interviewed, 14 (38%) were clinical officers, eight (22%) were HIV counsellors, seven (19%) were nurses, four (11%) were social workers and three (9%) were data clerks and a pharmaceutical technologist.

### 3.1 Adherence to PrEP delivery guidelines

**Figure 1:**
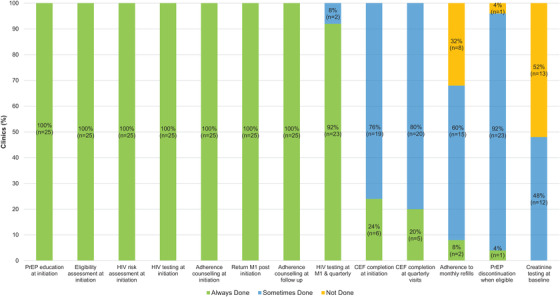
**Adherence to activities laid out in the Kenyan Ministry of Health PrEP service delivery guidelines**. CEF, clinical encounter form; M1, month 1; PrEP, pre‐exposure prophylaxis.

At the initial PrEP visit, all (*n* = 25) clinics provided HIV testing, one‐on‐one PrEP education, HIV risk and clinical eligibility assessment and adherence counselling prior to PrEP initiation (Figure 1). All clinics also asked clients to return one month after PrEP initiation for refills and review. However, creatinine testing was conducted by only 12 (48%) of the clinics some of the time and not at all by 13 (52%) of the clinics. Only six (24%) clinics reported completing clinic encounter forms fully all the time.

At follow‐up visits, all clinics conducted adherence counselling. Almost all clinics (*n* = 23, 92%) reported doing an HIV test at month 1 and then at quarterly visits consistently. One clinic reported not doing HIV testing at month 1, and another reported doing HIV testing at all visits including monthly refill visits. Both of these clinics later reverted to testing as prescribed in the guidelines. While the guideline stipulated that clients return to clinics for monthly PrEP refills, the majority of clinics issued more than one bottle of PrEP some (*n* = 15, 60%) or all (*n* = 8, 32%) of the time at follow‐up visits. Almost none of the clinics (*n* = 2, 8%) discontinued PrEP when their known HIV+ partner(s) attained viral suppression. The clinic encounter form was fully completed all the time at follow‐up visits in only five (20%) clinics.

### 3.2 Modifications to existing public HIV care clinic practices

**Figure 2:**
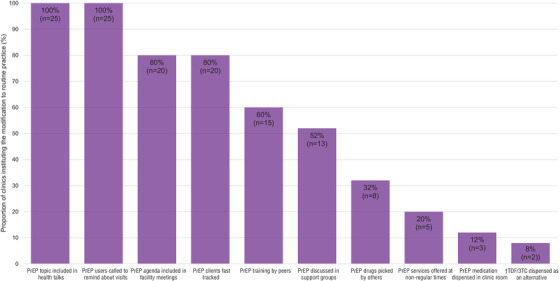
**Modifications made to existing HIV care programme practices to facilitate PrEP delivery in public HIV care clinics in Kenya**. PrEP, pre‐exposure prophylaxis; ^†^TDF/3TC, tenofovir disoproxil fumarate/lamivudine.

All clinics (*n* = 25) incorporated PrEP‐related topics in their regularly scheduled health talks (Figure 2 and Table 1). They also made phone calls to reach their PrEP clients who failed to attend scheduled visits. The majority of clinics included PrEP discussions in their routine facility meetings (*n* = 20, 80%) and also fasttracked PrEP clients (i.e., escorting them directly to the clinical room and pharmacy, so that they do not queue) (*n* = 20, 80%). Other frequently instituted practices included on‐the‐job training (OJT) by colleagues skilled in PrEP delivery (*n* = 15, 60%), including PrEP discussions at already existing ART support groups or at newly constituted PrEP groups (*n* = 13, 52%), providing PrEP services outside of regular clinic hours (*n* = 5, 20%) and dispensing PrEP medication in a clinical room rather than in pharmacy (*n* = 3, 12%).

**Table 1:** Adaptations to content in Kenyan Ministry of Health PrEP implementation guidelines (2018)^[^ ^25^ ^]^ and to the context in which PrEP services are offered in public HIV care clinics

Activity	Description	Description of adaptation	Nature of modification	Who participated in decision to modify	For whom the modification was made	Goal of modification
Creatinine testing	Creatinine testing is recommended at PrEP initiation, but absence of results should not delay PrEP initiation	No clinic did creatinine testing for all people initiating PrEP. Most clinics had some testing or no testing at all	Content modification with removal of elements	PrEP programme managers at national level Individual health provider	Health facility PrEP User	Increase PrEP uptake as creatinine tests are costly and not readily available
Frequency of PrEP refills	PrEP medication should be issued monthly	At quarterly visits most facilities issued PrEP medication that exceeded one‐month supply	Content modification with tailoring of elements	Individual health provider Health facility managers	PrEP User	Increase convenience for PrEP users Improve continuation
Discontinuation of PrEP	PrEP should be discontinued when HIV risk ends	PrEP is discontinued not only when there is no HIV risk but also when the PrEP user is ready to discontinue	Content modification with tailoring of elements	Individual health provider	PrEP User	Increase satisfaction among PrEP users
PrEP‐related health talks	Health talks on various topics are conducted at waiting bays routinely	Health facilities incorporated PrEP education in their health talks	Content modification with addition of elements	Health facility managers	Potential PrEP users Partners of potential PrEP users	Increase awareness about PrEP Improve PrEP uptake
Phone calls about scheduled appointments	Health workers routinely make phone calls to remind ART clients about their upcoming or missed appointments	Health workers included PrEP users in their list of individuals to be called	Content modification with addition of elements	Health facility managers Individual health provider	Health facility PrEP users	Improve PrEP continuation at facility level Improve adherence to scheduled visits
PrEP agenda included in facility meetings	Health facilities routinely held meetings to discuss HIV service delivery	PrEP service delivery was included as a discussion item in routine facility meetings	Content modification with addition of elements	Health facility managers	Health facility PrEP users	Improve service delivery Improve uptake Improve continuation
Fasttrack PrEP users	People at HIV clinics queue for their services, and fasttrack very sick patients	People initiating or continuing PrEP use do not wait in queues but are fasttracked through service delivery points	Contextual modification to delivery setting	Health facility managers Individual health provider	PrEP users	Reduce waiting time Reduce stigma Improve continuation Increase satisfaction
PrEP training by peers	On‐the‐job training by skilled peers for HIV treatment services	PrEP service delivery added as a skill to be trained by skilled peers	Content modification with addition of elements	Health facility managers Individual health provider	Individual health provider	Increase number of providers able to provide PrEP services Reduce workload
Support groups	Support groups are held among HIV‐positive persons and their partners to discuss living positively	PrEP incorporated in discussions held at support groups In some clinics, support groups specifically for PrEP users were set up	Content modification with addition of elements	Individual health provider Health facility managers	Potential PrEP users Partners of potential PrEP users PrEP users	Increase awareness about PrEP Improve PrEP uptake Improve PrEP continuation
Allow others to pick up drugs	Clients do PrEP refills in person	Health facilities allowed partners of PrEP users to pick medication for them when they could not come to the clinic	Contextual modification to format of delivery	Individual health provider	PrEP users	Improve PrEP continuation
PrEP delivery outside of regular clinic hours	PrEP services offered during regular hours	Some clinics offered PrEP services at nonregular clinic hours, for example, late afternoon or on days when the clinic is least busy	Contextual modification to delivery setting	Health facility managers	PrEP users	Improve PrEP continuation Reduce waiting time Reduce stigma Increase client satisfaction
PrEP dispensed in clinic rooms	PrEP medication dispensed in pharmacy	A few clinics dispensed PrEP medication in the clinic rooms rather than asking clients to get served in pharmacy	Contextual modification to delivery setting	Health facility managers	PrEP users	Improve PrEP continuation Reduce waiting time Reduce stigma Increase client satisfaction

*Note*: Description of the modifications is guided by the FRAME: an expanded framework for reporting adaptations and modifications to evidence‐based interventions [20].

### 3.3 PrEP uptake and continuation

**Figure 3:**
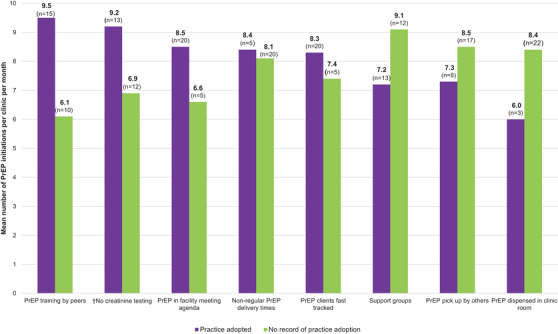
**Meanpre‐exposure prophylaxis (PrEP) initiations among clients at public HIV care clinics that adopted specified modifications compared to clients at clinics that did not**. Mean number of individuals initiating PrEP at facilities that did (purple) or did not (green) implement specified modifications. ^†^Creatinine: purple bar ‐ no creatinine testing done; green bar ‐ creatinine testing done sometimes.

By December 2018, 4136 people had initiated PrEP at an average of eight initiations per clinic per month and 55% of individuals were continuing to use PrEP six months after initiation across the 25 clinics. Clinics that did not require creatinine testing at PrEP initiation visits had higher mean monthly PrEP initiations compared to those that required testing some of the time (9.2 vs. 6.9 initiations per month) (Figure 3). Additionally, clinics that included PrEP in their meeting agenda (8.5 vs. 6.6), conducted peer PrEP training (9.5 vs. 6.1), and fasttracked PrEP clients (8.3 vs. 7.4) had higher mean monthly PrEP initiations compared to clinics in which there was no report of adoption of these practices.

**Figure 4:**
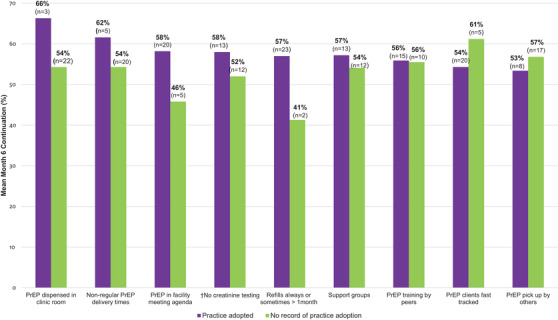
**Mean month 6 pre‐exposure prophylaxis (PrEP) continuation among clients at public HIV care clinics that adopted specified modifications compared to clients at clinics that did not**. Proportion of all individuals initiating PrEP who were continuing to use PrEP at six months in facilities that did (purple) or did not (green) implement specified modifications. ^†^Creatinine: purple bar ‐ no creatinine testing done; green bar ‐ creatinine testing done sometimes.

The mean six‐month continuation was higher among clinics that did not require creatinine testing (58% vs. 52%), included PrEP discussions in their meeting agenda (58% vs. 46%), had refill times greater than a month (57% vs. 41%), provided PrEP services outside of regular clinic hours (62% vs. 54%) and dispensed PrEP in clinic rooms (66% vs. 54%) (Figure 4).

### 3.4 Provider perspectives on modifications

PrEP providers described reasons for and successes resulting from the changes made to PrEP delivery guidelines and modifications made to routine clinic practices. These reasons are presented in four categories.

#### 3.4.1 To address client needs

Health providers often instituted practices to meet the needs of PrEP clients. They reported that PrEP users did not want to be seen in an HIV clinic due to the stigma associated with the clinics. Long waiting times in the facility were believed to increase the likelihood that they would be spotted by someone known to them, who would label them as living with HIV. To address this concern, health facilities instituted fasttracking, in which PrEP users did not queue but were escorted directly to the clinic and pharmacy.

[PrEP clients] feel the services are better [now] and they feel they are cared for as compared to the initial stages where we were not fasttracking. [Before] they felt they were stigmatized especially, you know, their neighbours…[who] are saying that this person is nowadays taking ARVS. But since we started fast tracking them, … they feel they are being cared for. (KII, Clinical Officer)

Other practices aimed at addressing waiting time and stigma associated with being in an HIV clinic included dispensing PrEP in clinic rooms rather than having them queue in pharmacy and providing services outside of regular clinic hours.

Another modification adopted to address client needs was continuing their PrEP prescriptions, even if their partners had attained viral suppression. Healthcare providers reported that PrEP users often did not want to give up their PrEP medication as they had no guarantee that their partners would remain virally suppressed.

They [PrEP clients] talk about the fear, they fear that they are being exposed and they can contract HIV. In fact, most of our clients, their partners are virally suppressed and they feel that they should not stop so you cannot force them, you can't force them to stop. (KII, HIV Testing Provider)

#### 3.4.2 To address provider needs

Clinics made adaptations to address challenges that providers faced, especially those related to increased workload. For example, if they were short‐staffed, some clinicians preferred to make side notes rather than spend time completing the entire client encounter form. In addition, because there were only a few providers who had attended formal PrEP trainings, health workers made efforts to train their peers how to provide PrEP services. OJT was instituted in some facilities upon realization that when those formally trained in PrEP were not available, PrEP eligible persons were sometimes unattended.

At first, because only two people had gone for the training, so it was difficult… … [T]he clinical officer and the records officer were the [only] ones issuing PrEP but now an OJT was done and all of us as the clinical officers, we were updated about PrEP. So now it is on a rotational basis. Everyone does that [PrEP] delivery per week, so it is easier now than before. (KII, Clinical Officer)

#### 3.4.3 To improve PrEP uptake

Most health facilities opted to not require creatinine tests before PrEP initiation as many clients could not afford to do them. Although providers often stated that they would like to have creatinine test results, they knew that if they insisted on having this test, many people would leave without initiating PrEP.

In all HIV clinics, topics related to PrEP were introduced in routinely conducted health talks in order to increase PrEP awareness among people living with HIV and encourage them to bring in their partners to receive PrEP.

One of the things that we do to create demand creation in the facility is to give effective health talks in the facility. We are able to educate our clients on PrEP, the eligibility criteria for PrEP, and that makes people come for PrEP. (KII, Social Worker)

#### 3.4.4 To improve PrEP continuation

Many clinics reported having meetings in the facility during which they discussed strategies to improve PrEP continuation. For example, shortly after starting PrEP services, health providers began to notice that their clients were not returning as scheduled and they began calling them, as they do in HIV treatment programmes.

Literally we would just leave them [PrEP clients] to come and when we realize a patient has not come, we start doing follow‐up. So, we have decided to be calling them two days prior to their clinic day. (KII, HIV Testing Provider)

Many clinics issued more than one bottle at quarterly visits, when they realized PrEP users failed to come for refills because frequent visits posed a burden and interfered with their day‐to‐day activities. Having longer refill dates was reported as welcomed by PrEP users, as well benefitting health providers by reducing workload.

If you give them a shorter TCA, let's say one month, they will not be around to pick their drugs. So, if you give them longer TCAs according to their convenience, then it will always improve their retention. (KII, Nurse)

## 4 DISCUSSION

Health providers made innovative adaptations to activities detailed in PrEP guidelines and instituted modifications to routine practices. These changes were made to simplify PrEP delivery processes for clients and to reduce service delivery barriers among health workers.

Public HIV care clinics were adherent to most of the requirements laid down in the guidelines, including HIV testing, behavioural risk assessment, PrEP education and adherence counselling. While creatinine testing was required, there was no clinic that conducted creatinine testing all the time at PrEP initiation, possibly because guidelines advised that lack of creatinine results should not delay initiation. All facilities made modifications to some of their existing HIV care programme practices in order to improve the ease with which clients obtain PrEP from HIV care clinics and to facilitate the integration of PrEP service delivery with available human resources and existing infrastructure.

Clinics that instituted contextual modifications such as PrEP discussions in routine facility meetings, on‐the‐job PrEP training by peers, multi‐month scripting and providing PrEP services outside of regular clinic hours had higher than average monthly PrEP initiations and continuation rates. The Partners Scale‐Up Project, which to our knowledge was the first evaluation of a national PrEP rollout in Africa, had a mean monthly PrEP uptake that was similar to that observed in similar settings, though we observed higher mean month 6 continuation rates [30, 31, 32].

Adaptations to activities laid out in PrEP guidelines and modifications to routine HIV clinic service delivery practices were aimed at strengthening a client‐centred delivery approach. In other studies, long waiting times, frequent visits (that interfere with work schedules and have associated transport costs), stigma and lack of confidentiality have been identified as barriers to engagement in care [33, 34, 35]. Client‐centred approaches directed at reducing these barriers and maximizing convenience and responsiveness to client needs and preferences have resulted in continued engagement in care and improved patient outcomes in HIV treatment programmes [36, 37]. In our project, health facilities similarly made modifications to their existing practices to reduce time spent seeking PrEP services and address stigma. Additionally, providers accommodated user preferences by not insisting on PrEP discontinuation even when their known partners living with HIV attained viral suppression. Client‐centred approaches for HIV prevention programmes, including PrEP, have the potential to ensure increased access and efficacious use of HIV prevention strategies [38]. PrEP training curricula may be revised to inform health workers of adaptations made to PrEP service delivery so that they can consider incorporating such practices in their own clinics.

Health facilities instituted practices to lessen the effects of increased workload resulting from integrating PrEP delivery, such as training additional staff members and reducing the frequency of refill visits. Additional strategies to reduce workload burden and address staff shortages common in public health facilities might include task‐shifting, use of lay health workers and tele‐consultations [39, 40, 41, 42]. Cost‐effective training approaches such as OJT by peers can increase the number of health workers able to provide PrEP services and in this way address shortages of PrEP‐skilled staff [43, 44, 45]. Completion of programme records should be simplified and standardized and inefficiencies in the data collection system should be eliminated [46]. Developing electronic medical records for the PrEP programme may improve PrEP data collection systems and enhance data quality in public health facilities [47, 48].

Adaptability of an intervention is a key determinant of successful intervention implementation and scale‐up [49, 50, 51, 52]. Therefore, there is need to advance our understanding of intervention adaptations using data from real‐world practice ‘where the rubber meets the road’ in order to facilitate adoption and widespread implementation [14]. Research reporting on the full range of modifications and adaptations made to interventions in routine care settings is limited [14, 53]. In this study, we used FRAME to comprehensively characterize adaptations to PrEP implementation guidelines and to existing HIV care programme practices in order to facilitate PrEP implementation in public HIV care clinics [20]. Using this framework ensured that elements of the adaptations were examined and reported systematically. An important attribute of the FRAME is its flexibility, which enabled reporting aspects of modifications and adaptations that were relevant and applicable to this intervention. For example, the ‘adaptation timing’ element of the FRAME was not relevant in our study as all modifications were instituted post implementation. Reporting modifications within this framework may be especially useful to others scaling up PrEP programmes, as it enables an interrogation of the nature, context and goal of adaptations in detail.

A limitation of this study is that providers could have instituted changes in their practices and failed to mention it in the interviews. We endeavoured to mitigate this limitation through triangulation of key informant interviews with different cadres and with TA reports. Another limitation is the observational and descriptive nature of our study, which limited our ability to draw conclusions about causal effects of observed adaptations and modifications on PrEP uptake and continuation.

## 5 CONCLUSIONS

During the early years of PrEP scale‐up in Kenya, health providers in public HIV care clinics instituted practices and made innovative adaptations to PrEP delivery. Providers made these changes with the intent of improving ‘fit’ for PrEP users and providers and improving PrEP uptake and continuation. Encouraging clinic‐level adaptations to national implementation guidelines may facilitate scale‐up of PrEP delivery, and these findings may inform PrEP delivery in other health facilities in the country and programmes in the region.

## COMPETING INTERESTS

The authors declare that they have no competing interests.

## AUTHORS’ CONTRIBUTIONS

E.M.I. and G.O. conducted the analysis and wrote the first draft of the manuscript. J.M.B., N.R.M. and E.A.B. designed and led the project. J.O. and E.W. oversaw implementation in study sites. J.M.B., K.N., K.K.M. and J.F.M. critically reviewed the manuscript. All authors read and approved the final manuscript.

## FUNDING

The study was funded by the National Institute of Mental Health of the US National Institutes of Health(Grant Number R01MH095507) and the Bill & Melinda Gates Foundation(Grant Number OPP10556051).
