# Supplementary material for: Process evaluation of PrEP implementation in Kenya: adaptation of practices and contextual modifications in public HIV care clinics
Source: J Int AIDS Soc. 2021 Sep 8;24(9):e25799. doi: 10.1002/jia2.25799 (PMC8425783; doi:10.1002/jia2.25799)
Supplement: Supplementary file 1 [file JIA2-24-e25799-s003.docx]

1. General Information

| Site Name |  | |
| --- | --- | --- |
| Date of Visit |  | |
| Technical advisor(s) conducting the visit | Name | Title/Designation |
|  |  |  |
|  |  |  |
|  |  |  |
| Site staff who participated in the visit | Name | Title/Designation |
|  |  |  |
|  |  |  |
|  |  |  |
|  |  |  |
|  |  |  |

1. Support Visit Summary

Always try to complete status and comments section. Comments may include strengths and/or challenges. If you do not have time or are not able to assess state ‘not able to assess”. In the column for Support/Training provided, write n/a if no support was provided or no action points agreed upon for the corresponding component during this visit.

| **Component** | **Status and comments** | **Support/training provided during visit** |
| --- | --- | --- |
| Awareness & Demand Creation |  |  |
| Identification of PrEP Users |  |  |
| Provision of PrEP |  |  |
| Monitoring & Follow-Up |  |  |
| Workforce and Infrastructure |  |  |
| Commodities Management |  |  |
| Laboratory Systems |  |  |
| Monitoring and Evaluation |  |  |

**Action Plan**

Maintain an on-going action plan for each site. When you arrive for follow-up visits, refer to the previously entered challenges/issues, discuss the implementation status of the agreed action points with CCC management and staff, and document the process accordingly. Limit the active challenges/issues being addressed to a feasible and reasonable number in agreement with CCC staff.

| **Challenge/Issue** | **Action Point** | **Person(s) Responsible** | **Timeline** | **Status (date)** |
| --- | --- | --- | --- | --- |
|  |  |  |  |  |
|  |  |  |  |  |
|  |  |  |  |  |
|  |  |  |  |  |
|  |  |  |  |  |
|  |  |  |  |  |
|  |  |  |  |  |
|  |  |  |  |  |
|  |  |  |  |  |
