# Supplementary material for: Process evaluation of PrEP implementation in Kenya: adaptation of practices and contextual modifications in public HIV care clinics
Source: J Int AIDS Soc. 2021 Sep 8;24(9):e25799. doi: 10.1002/jia2.25799 (PMC8425783; doi:10.1002/jia2.25799)
Supplement: Supplementary file 2 [file JIA2-24-e25799-s001.docx]

**An implementation project to scale-up delivery of antiretroviral-based HIV-1 prevention**

**among Kenyan HIV-1 serodiscordant couples**

Qualitative component: To gain a deeper understanding of acceptance, barriers, facilitators, and opportunities for efficiency for delivery of PrEP.

***Target group*:** Health providers

*Objectives*:

- Describe PrEP delivery process and integration into the ART clinics
- Describe the impact of PrEP delivery to couples and health facilities
- Document suggestions from providers on opportunities for efficiency in PrEP delivery

*Demographic questionnaire*

|  | **Question** |
| --- | --- |
|  | *For Interviewer: Is the participant male or female?*  *____ Male ____Female* |
|  | What is your educational background? (e.g. nurse, doctor, clinical officer, pharmacist)  _____________________________________ |
|  | What is your current position? |
|  | How long have you been in your position? |
|  | How old are you? ___________ years |

# In depth Interview guide:

## **INTRODUCTION**

Welcome to our discussion. Please feel free to share anything you want with me.

## **TOPIC 1: PrEP DELIVERY process**

**What has been your experience in delivering PrEP in your clinic:**

Possible probes:

- Please tell us about your specific role/s

*(Probe further on the role of the participant and let him/her describe her/his roles in in PrEP delivery e.g. opening files, counseling, dispensing drugs, health talks, facilitating support groups etc.)*

- How is PrEP delivery & integration being implemented at this clinic?

*(Let them* explain *the delivery process- what happens when a client walks in until that time he/she walks out; ask initial visits and follow up visits separately, patient flow & perceived workload)*

- - Probe how and why the delivery method changed (evolved) since the clinic has been offering PrEP for X months.
- What has changed in your clinic since you started providing PrEP?
  - *Positive attributes? E.g. more couples coming? Better retention or adherence for the HIV infected.*
  - *Workload (explore how the workload has changed)*
  - *Waiting time (Probe if the introduction of PrEP has affected the waiting time of ART clients and/or PrEP clients, ask about fast tracking and its implication to deliver and uptake)*
  - What do your HIV uninfected clients tell you about their experiences of getting services in a HIV clinic?
  - What has been your experience in completing the clinic encounter form?
- How did you learn to deliver PrEP? Was that training adequate? Did you feel well prepared to deliver?Suggestions for improvement?
- What aspects of your clinic promote PrEP delivery? (regular meetings – is PrEP explicitly discussed, multi-disciplinary team)
- What aspects of your clinic hinder efficient delivery of PrEP ?
- What would you advice other health providers planning to start offering PrEP in their clinics to do (and not to do) to improve delivery?

## **TOPIC 2: STAFFING/PROVIDERS**

- Who is the person/s most involved with PrEP promotion in this clinic *(champions)*? What is their position**?** *What do they do to promote PrEP? How did this person/s become a champion?*
- Who are the persons who support the delivery process and what is their roles?
  - In-clinic staff
  - Out of clinic staff CASCOs/SASCO/TAs
    - What do they exactly do?
    - What would you wish them to do?
- Describe scenarios where a provider/s negatively influence the delivery process?

## **Topic 3: EXPERIENCE OF PROVIDING SERVICES TO COUPLES**

**What has been the impact of PrEP on serodiscordant couples?**

Possible probes:

- Do clients come as couples to your clinic? Why do some clients come as couples and why do some come as individuals? Clinic based and individual reasons
- In what ways has PrEP provision been beneficial to the HIV+ partners (adherence, retention, viral suppression, safer conception, disclosure, partner testing)

## **Topic 4: DEMAND CREATION FOR PReP**

- What is your facility doing to identify people for PrEP delivery (demand creation strategies)?
  - Which of these strategies is most successful to get people to initiate PrEP?
  - What have you tried and did not work
- What else could be done to improve recruitment?
- What are some issues that clients have raised that promote their demand creation?

## **Topic 5: PREP ADHERENCE**

- Tell me how your clinic promotes PrEP adherence?
  - What works to promote adherence for persons taking PrEP in your clinic?
  - What have you tried and did not work?
- What else could be done to improve adherence?
- what are some issues that clients have raised that promote their adherence?

## **Topic 6: RETENTION for clients on prep**

- Tell me how your clinic retains PrEP clients?
  - What works to promote retention for persons taking PrEP in your clinic?
  - What have you tried and did not work?
  - What else could be done to improve retention?
- What are some issues that clients have raised that promote their retention?
- What are the barriers to retention for the HIV- on PrEP in your clinic? (E.g. short TCA, lack of bus fare, time, lost cards, forgot appointment date etc..)

## **Topic 7: COUNSELLING**

**When discussing PrEP with clients are there particular messages that seem most useful?**

**How do you explain PrEP to couples?**

*(Let the HCWs give example of those messages verbatim e.g. I tell them “PrEP is only one pill per day”)(If possible do a role play and ask the HCW to assume you are a client and tell you what he/she tells or would tell a client) (Ask separately for initiation and continuation)*

Possible probes:

PrEP Efficacy

- What are your feelings about how well PrEP works? And what do you tell clients on how well PrEP works? What do you tell clients on how soon PrEP starts to work?
- How easy or difficult is it to counsel clients on PrEP?

Condoms

- What do you think regarding use of condoms with PrEP?
- What do you tell clients regarding condoms in the context of PrEP(Also could be: What do clients ask you about condoms? What do you tell them? Or What do clients say when you talk to them about condoms in the context of PrEP?)

PrEP Discontinuation

- When do you think it’s the right time to discontinue PrEP?
- In what situations do PrEP users continue on PrEP even after the HIV+ partner has achieved viral suppression?
- What do you tell clients regarding PrEP discontinuation? How acceptable is PrEP discontinuation?
- What are the clients saying regarding PrEP discontinuation?

Viral suppression

- What are your feelings about how well ART works as HIV prevention? And what do you tell clients on how well ART for HIV prevention works? What do you tell clients on how soon ART starts for HIV prevention?

## **Topic 8: Closing discussion**

***Any other thoughts or suggestions on PrEP delivery that you would like to share?***
