# Supplementary material for: Process evaluation of PrEP implementation in Kenya: adaptation of practices and contextual modifications in public HIV care clinics
Source: J Int AIDS Soc. 2021 Sep 8;24(9):e25799. doi: 10.1002/jia2.25799 (PMC8425783; doi:10.1002/jia2.25799)
Supplement: Supplementary file 3 [file JIA2-24-e25799-s002.docx]

Participating clinics and project team

**Participating HIV care clinics**

1. Ahero Sub County Hospital
2. Bondo Sub County Hospital
3. Coptic Hope Center, Ngong road
4. Coptic Medical Center, Industrial Area
5. Karatina Sub-County Hospital
6. Kerugoya County Referral Hospital
7. Kiambu County Referral Hospital
8. Kisumu County Referral Hospital
9. Kombewa Sub County Hospital
10. Lumumba Sub County Hospital
11. Mbagathi County Hospital
12. Migori County Referral Hospital
13. Muhoroni Sub County Hospital
14. Murang'a County Referral Hospital
15. Nyakach Sub County Hospital
16. Nyeri County Referral Hospital
17. PCEA Kikuyu Hospital
18. Pumwani National Referral Maternity Hospital
19. Rabuor Sub County Hospital
20. Rachuonyo Sub County Hospital
21. Rongo Sub County Hospital
22. Ruiru Sub-County Hospital
23. Siaya County Referral Hospital
24. St. Mary’s Mission Hospital
25. Thika Level V Hospital

**Partners Scale-Up Project Team**

Western Region:

Elizabeth Bukusi (Principal Investigator), Josephine Odoyo (coordinator), Benjamin Angasa, Magdalene Asewe, Merceline Awuor, Annabell Dollah, Sylvia Mugalla, Bernard Nyerere, Wycliff Odhiambo, Joel Odondi, Meresa Oyier, John Bosco Tsetso

Central Region:

Nelly Mugo (Principal Investigator), Elizabeth Wamoni (coordinator), Victoria Kyengo, Margaret Mwangi, Rosemary Ngacha, Roy Njiru, Fernandos Ongolly, Cyrus Theuri, Winnie Waituika, Irene Wanyoike

Coordinating Centre – University of Washington, Seattle:

Jared Baeten (Principal Investigator), Elizabeth Irungu, Lara Kidoguchi, Jennifer F. Morton, Kenneth Mugwanya, Kenneth Ngure, Gabrielle O’Malley, Sue Peacock
